# Lymphocyte Count, Serum Albumin and Transferrin Levels in Patients Undergoing Total Knee Arthroplasty

**DOI:** 10.1055/s-0045-1809529

**Published:** 2025-07-25

**Authors:** Bernardo Crespo Alves, Amanda S. Cavalcanti, Kelly Biancardini Gomes Barbato, João Maurício Barretto, Juliana Arruda de Matos

**Affiliations:** 1 Centro de Atenção Especializada ao Joelho, Instituto Nacional de Traumatologia e Ortopedia, Rio de Janeiro, RJ, Brasil; 2 Hospital Clementino Fraga Filho, Universidade Federal do Rio de Janeiro (UFRJ), Rio de Janeiro, Brasil; 3 Divisão de Pesquisa, Instituto Nacional de Traumatologia e Ortopedia, Rio de Janeiro, RJ, Brasil; 4 Grupo de Joelho do Hospital São Vicente, Rede D'Or, Rio de Janeiro, RJ, Brasil; 5 Comissão de Controle de Infecção Hospitalar, Instituto Nacional de Traumatologia e Ortopedia, Rio de Janeiro, RJ, Brasil

**Keywords:** knee, osteoarthritis, arthroplasty, replacement, knee, malnutrition, periprosthetic joint infection, joelho, osteoartrite, artroplastia do joelho, desnutrição, infecção articular periprotética

## Abstract

**Objective:**

Describe the prevalence of preoperative malnutrition in individuals undergoing primary TKA and to assess its association with age, sex, body mass index (BMI), and comorbidities, as well as the risk of prolonged postsurgical hospitalization, early prosthetic joint infection (PJI), or readmission.

**Methods:**

We conducted a cohort study of TKAs performed between 2014 and 2016. Preoperative malnutrition was defined as a total lymphocyte count < 1,500 cells/mm ^3^ , a serum albumin concentration < 3.5 g/dL, or a transferrin concentration < 200 mg/dL within the six months before surgery.

**Results:**

Out of the 2080 TKAs performed, 1099 had valid lymphometry, albumin, and transferrin data and were included in the analysis. The prevalence of malnutrition was 17.7%. Independent factors associated with a higher prevalence of malnutrition were age (OR = 1.03; 95% CI = (1.01–1.05)), anemia (1.55 (1.05–2.28)), low weight (3.13 (1.50–6.50)), and normal weight (1.85 (1.21–2.82)). Diabetes mellitus was inversely associated with malnutrition (0.60 (0.38–0.96)). Early PJI was diagnosed in 18 (1.6%) participants. There was no statistically significant and independent association between malnutrition and postsurgical complications.

**Conclusion:**

Altered lymphocyte count, serum albumin, and transferrin levels is common among individuals undergoing TKA, particularly in older patients, those with anemia, and individuals with normal or low weight. Future studies with larger sample sizes are needed to better assess the relationship between malnutrition and adverse outcomes following TKA.

## Resumo

**Objetivo:**

Descrever a prevalência de desnutrição pré-operatória em indivíduos submetidos à ATJ primária e avaliar sua associação à idade, sexo, índice de massa corporal (IMC) e comorbidades, bem como o risco de hospitalização pós-operatória prolongada, infecção articular periprotética (IAP) precoce ou readmissão.

**Métodos:**

Este é um estudo de coorte de ATJs realizadas entre 2014 e 2016. A desnutrição pré-operatória foi definida pelo número total de linfócitos < 1.500 células/mm ^3^ , concentração sérica de albumina < 3,5 g/dL ou concentração de transferrina < 200 mg/dL nos 6 meses anteriores à cirurgia.

**Resultados:**

Das 2.080 ATJ realizadas, 1.099 casos tinham dados válidos de linfometria, albumina e transferrina e foram incluídas na análise. A prevalência de desnutrição foi de 17,7%. Os fatores independentes associados à maior prevalência de desnutrição foram idade (razão de chances [OR] = 1,03; intervalo de confiança [IC] de 95% = 1,01-1,05), anemia (1,55 [1,05-2,28]), baixo peso (3,13 [1,50-6,50]) e peso normal (1,85 [1,21-2,82]). O diabetes mellitus foi inversamente associado à desnutrição (0,60 [0,38-0,96]). A IAP precoce foi diagnosticada em 18 (1,6%) participantes. Não houve associação estatisticamente significativa e independente entre desnutrição e complicações pós-cirúrgicas.

**Conclusão:**

As alterações em números de linfócitos e concentrações séricas de albumina e transferrina são comuns em indivíduos submetidos à ATJ, em especial em pacientes mais velhos, anêmicos e com peso normal ou baixo. Estudos futuros com amostras maiores são necessários para avaliar melhor a relação entre desnutrição e desfechos adversos após ATJ.

## Introduction

Malnutrition is an imbalance in caloric, protein, or vitamin intake that leads to acute or chronic nutritional depletion and associated metabolic or functional impairments. 1 This condition may arise from reduced food intake, absorptive disorders, hypercatabolic states, or consumptive conditions. 2 Various diagnostic criteria, including anthropometric indices (e.g., body mass index and body composition analysis), nutritional questionnaires, and serum markers, are used to define malnutrition, each with its own limitations and advantages.

Despite the availability of various methods for screening and assessing malnutrition—such as anthropometric measurements, body composition analysis, nutritional questionnaires, and laboratory markers—its diagnosis remains imprecise. The World Health Organization (WHO) defines malnutrition as a BMI below 18.5 kg/m ^2^ . In contrast, the American Society for Parenteral and Enteral Nutrition (ASPEN) defines it based on the presence of two or more criteria, including inadequate caloric intake, weight loss, loss of muscle mass, loss of subcutaneous fat, localized or generalized fluid accumulation that may mask weight loss, and decreased functional capacity. 2 The European Society for Clinical Nutrition and Metabolism (ESPEN) has proposed a consensus for diagnosing malnutrition, which combines the use of nutritional risk questionnaires with criteria such as BMI, relative weight loss, or lean mass index. 3 This consensus has been criticized for its impracticality, lack of clear guidance on which nutritional risk questionnaire to use, and its neglect of inflammatory biochemical changes involved in the malnutrition process. 4 5 Serum markers are widely used for nutritional assessment due to their affordability, reproducibility, and ability to detect acute changes in nutritional status. Common parameters for identifying malnutrition include a total lymphocyte count below 1,500 cells/mm ^3^ , serum albumin levels under 3.5 g/dL, and transferrin levels below 200 mg/dL. 6 Other nutritional markers, such as prealbumin, interleukins, C-reactive protein, and leptin, are less frequently utilized. 7

The link between malnutrition and postoperative complications was first noted in abdominal surgeries. 8 In orthopedics, malnutrition has been associated with poorer outcomes in patients treated for proximal femur fractures, leading to longer hospital stays, increased infectious and noninfectious complications, and higher readmission rates. 9 10

Total knee arthroplasty (TKA) is a complex surgery aimed at treating advanced knee osteoarthritis to restore function and relieve symptoms. Projections indicate a rising demand for arthroplasties, significantly impacting public and private healthcare costs. 11 Prosthetic joint infection (PJI) is the leading cause of early failure and short-term revision surgery. 12 Research on the link between malnutrition and PJI, as well as noninfectious complications in arthroplasties, is gaining attraction in the literature. 13 14 However, Brazilian data are scarce, highlighting the need to evaluate nutritional status to understand its impact and develop strategies for risk mitigation.

This study aimed to assess the prevalence of preoperative malnutrition and its association with demographic and clinical characteristics, as well as the risk of PJI and noninfectious complications post-TKA in a reference orthopedic surgery center.

## Materials and Methods

This cohort study involved a retrospective analysis of prospectively collected data, approved by the Institutional Review Board (CAAE 63639216.3.0000.5273). It included all patients who underwent primary TKA from January 2014 to December 2016. Patients who had simultaneous bilateral arthroplasties or resided outside Rio de Janeiro State were excluded.

All surgeries were performed by specialist knee surgeons. Patients received antimicrobial prophylaxis 30 to 60 minutes before the incision and were transferred to the intensive care unit on the first postoperative day. Hospital discharge occurred from the third day post-admission, based on adequate clinical conditions and pain control. Thromboembolic event prevention was performed with a daily single subcutaneous dose of 40 mg of low-molecular-weight heparin (Clexane®, Sanofi Aventis), starting 12 to 24 hours after surgery and continuing for 14 days. Postoperative patient follow-up was conducted through regular outpatient consultations.

Deep PJI diagnosis followed the Musculoskeletal Infection Society (MSIS) guidelines. 15 PJI was classified temporally based on the interval from the index procedure to symptom onset: early (<3 months), intermediate (3 months to 2 years), or late (>2 years). 16 Only early infections were analyzed due to their direct association with perioperative risk factors.

Malnutrition was defined by at least one of the following laboratory parameters: total lymphocyte count <1,500 cells/mm ^3^ , serum albumin <3.5 g/dL, or transferrin <200 mg/dL. 6 For patients over 60, BMI was categorized per Pan American Health Organization (PAHO) recommendations: underweight (<23 kg/m ^2^ ), normal (23 to 27.9 kg/m ^2^ ), overweight (28 to 29.9 kg/m ^2^ ), and obese (>30 kg/m ^2^ ). 17 For those under 60, BMI was categorized using modified WHO classifications: underweight (<18.5 kg/m ^2^ ), normal (18.5 to 24.9 kg/m ^2^ ), overweight (25 to 29.9 kg/m ^2^ ), or obese (>30 kg/m ^2^ ). Anemia was defined per WHO criteria based on sex: hemoglobin <12 g/dL for females and <13 g/dL for males. 18

The hospital database was searched for information on all patients who underwent primary TKA between 2014 and 2016. Data collected included age, sex at birth, medical records, weight, height, comorbidities (such as hypertension, diabetes, asthma, heart disease, dyslipidemia, and rheumatoid arthritis), ASA classification, surgery date, admission and discharge dates, reason for discharge, perioperative blood transfusion, and preoperative laboratory results (hemoglobin, hematocrit, albumin, transferrin, and lymphocytes). Valid laboratory tests were those performed within 180 days before surgery; if multiple results existed, the closest to the surgery date was used for analysis. New admissions within 90 days post-procedure were reviewed through physical medical records. Additionally, epidemiological surveillance records from the Hospital Infection Control Committee were utilized to identify patients with early PJI. Data from both sources were entered into a Microsoft Excel 2016® spreadsheet.

Quantitative variables are presented as medians with interquartile ranges (IQRs), minimums, and maximums, while categorical variables are expressed as frequency (n) and percentage (%). The Shapiro—Wilk test assessed sample normality. For normally distributed data, the parametric Student's *t* -test was used, with adjustments for unequal variances as needed. For non-normal data, the Mann—Whitney or Kruskal—Wallis tests were employed. Categorical variables were analyzed using the chi-square or Fisher's exact tests, as appropriate. Odds ratios assessed the association between malnutrition and explanatory variables. Proportional hazard regression analyzed postoperative hospitalization time, treating time as a discrete variable due to its non-normal distribution ( *pgmhaz* command). Logistic regression identified independent factors associated with malnutrition. The McNemar test compared malnutrition prevalence based on different criteria. Statistical significance was set at 5% ( *p* < 0.05), and data were analyzed using Stata® version 17.0 (StataCorp LLC, College Station, Texas, USA).

## Results

**Fig. 1:**
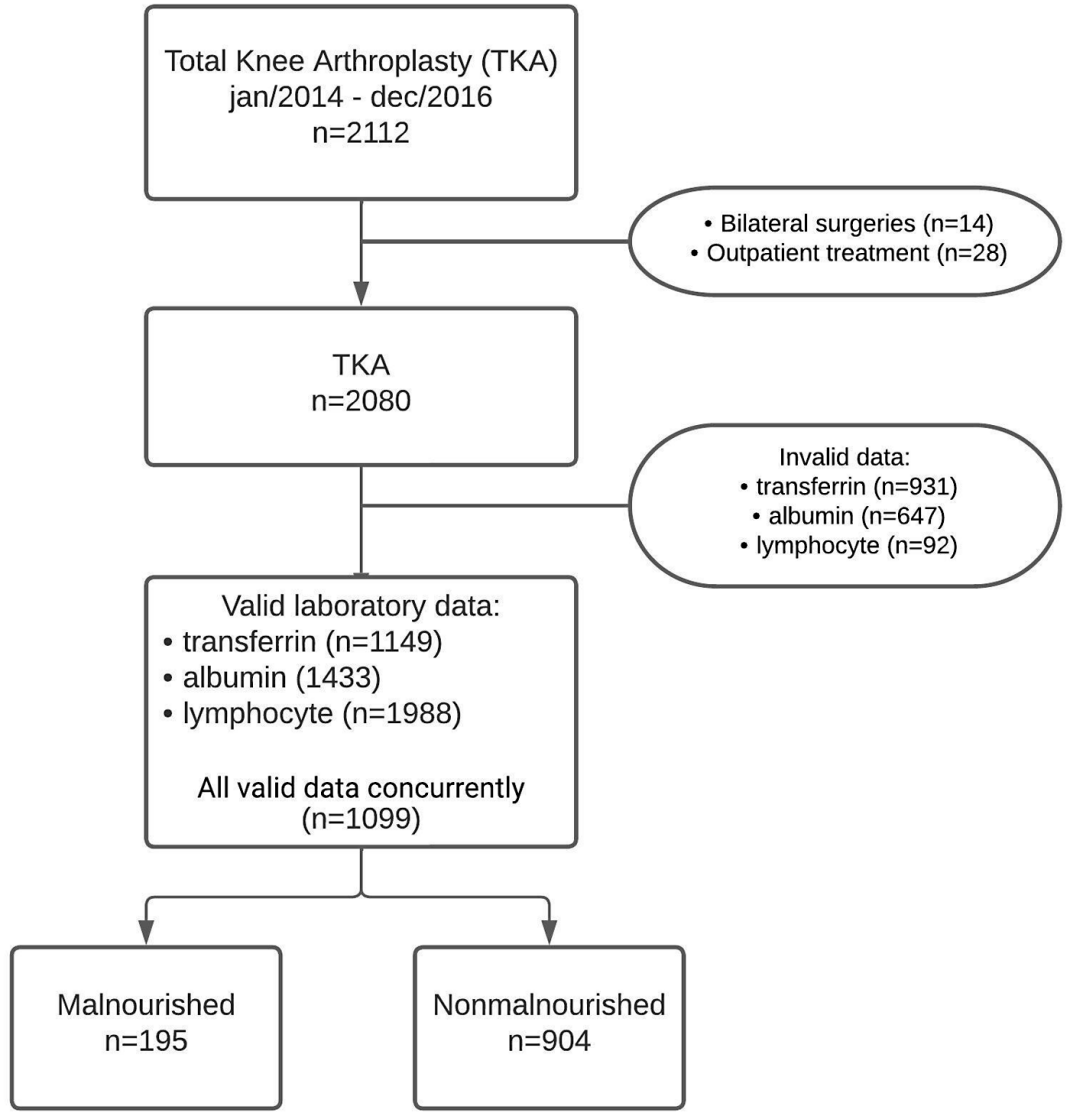
Flowchart of inclusion of participants in the study.

Between January 1, 2014, and December 31, 2016, a total of 2,112 primary TKAs were performed. After applying exclusion criteria (14 simultaneous bilateral TKAs and 28 patients from outside Rio de Janeiro State), 2,080 TKAs were analyzed ( Fig. 1 ). Of these, 73.6% were female, with a median age of 68 years (IQR 63–74). Complete patient data are provided in Supplementary Table S1 .

**Fig. 2:**
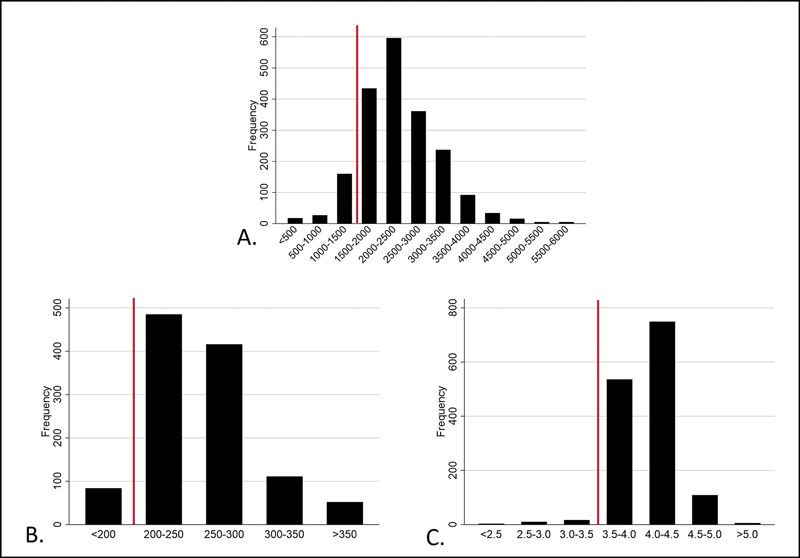
Distribution of laboratory markers of malnutrition, measured up to 180 days before primary total knee arthroplasty. The red line on each graph denotes the cutoff point for the malnutrition criterion. 3 A. Lymphocyte count ( *n* = 1,988): 205 participants with dosage below 1,500 cells/mm3; B. Transferrin dosage ( *n* = 1,149): 84 participants with dosage below 200 mg/dL; C. Albumin dosage ( *n* = 1,433): 30 participants with dosage below 3.5 g/dL.

Valid results for serum albumin, total transferrin, and total lymphocyte counts were obtained for 1,433, 1,149, and 1,988 participants, respectively. The median serum albumin was 4.0 mg/dL (IQR 3.8–4.2), with 30 patients (2.1%) exhibiting hypoalbuminemia (<3.5 g/dL). The median transferrin concentration was 250 mg/dL (IQR 226–279 mg/dL), and 84 patients (7.3%) had transferrin levels <200 mg/dL. The median total lymphocyte count was 2,288 cells/mm ^3^ (IQR 1,870–2,834), with 205 patients (10.3%) below 1,500 cells/mm ^3^ ( Fig. 2 and Supplementary Table S2 ).

**Fig. 3:**
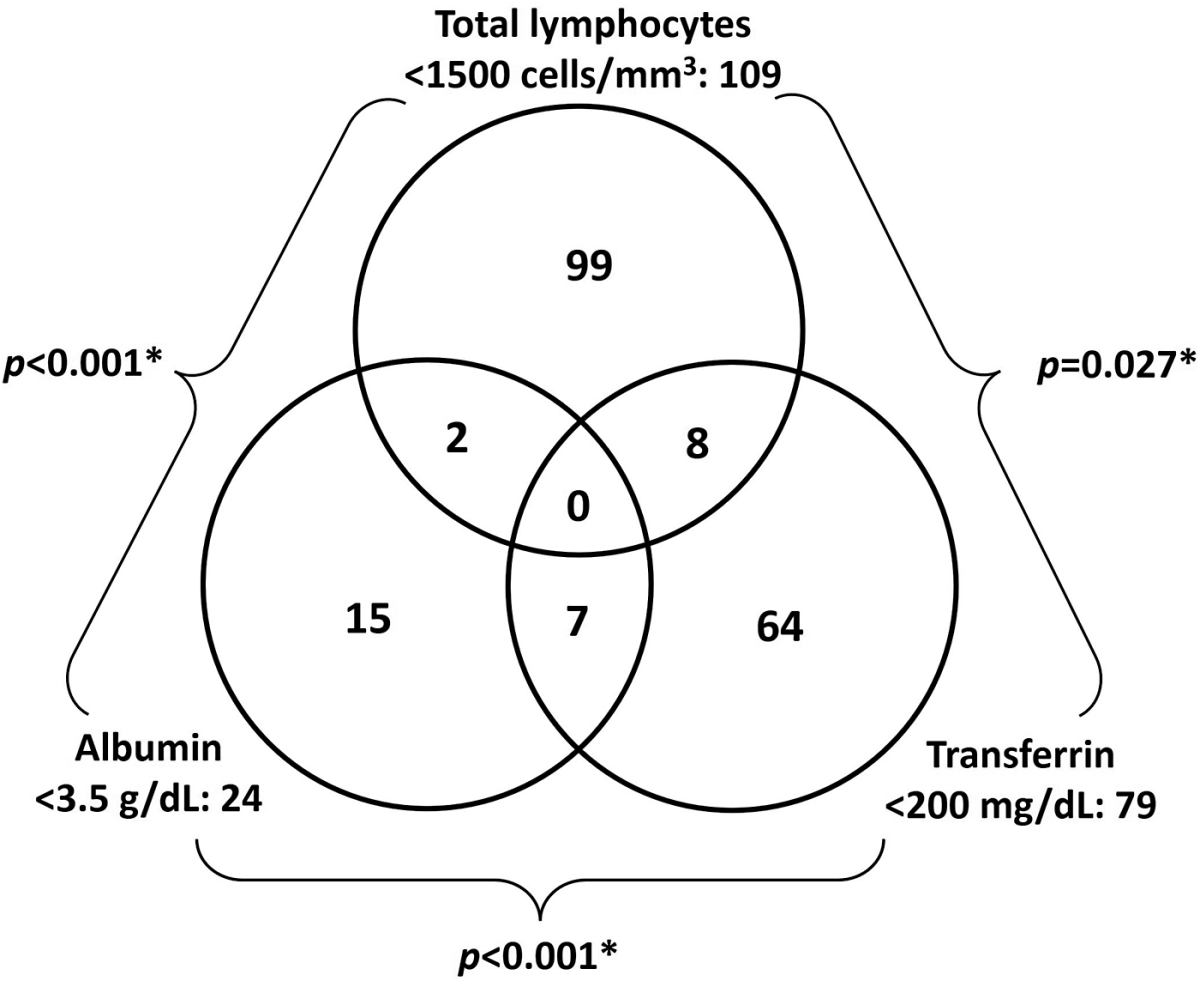
Distribution of the 195 patients classified as malnourished (among the 1,099 with the 3 available and valid laboratory parameters), according to the laboratory-defined malnutrition.

Out of 2,080 participants, 1,099 had valid data for albumin, transferrin, and lymphocytes, with 195 categorized as malnourished (17.7%) and 904 as nonmalnourished (82.3%). Among the malnourished, 17 individuals (8.7%) showed concurrent abnormalities in two or more serum markers. Pairwise concordance analysis revealed a statistically significant difference ( *p* < 0.05), indicating low overlap among these markers ( Fig. 3 ).

Malnourished individuals were older, had lower hematocrit and leukocyte counts, and lower BMI than their nonmalnourished counterparts. The prevalence of males was higher among the malnourished (p > 0.05). Malnourished individuals also had higher rates of underweight and normal weight classifications, greater prevalence of anemia, and more frequent blood transfusions compared with the nonmalnourished. Hypertension and diabetes were more common among individuals without laboratory-defined malnutrition ( Table 1 ).

**Table 1:** Analysis of demographic and laboratory characteristics between malnourished and non-malnourished patients undergoing primary total knee arthroplasty ( *n* = 1099)

	Not Malnourished ( *n* = 904)		Malnourished ( *n* = 195)		Total		Odd Ratio	95% CI	p-valor
Gender – N(%)									
Female	678	(75.0%)	133	(68.2%)	811	(73.8%)			
Male	226	(25.0%)	62	(31.8%)	288	(26.2%)	1.40	(1.00, 1.96)	0.051
BMI (OMS/OPAS)– N(%)									
Obesity	480	(57.7%)	73	(42.2%)	553	(55.0%)	1.00	–	–
Overweight	139	(16.7%)	33	(19.1%)	172	(17.1%)	1.56	(0.99, 2.45)	0.054
Normal Weight	188	(22.6%)	53	(30.6%)	241	(24.0%)	1.85	(1.25, 2.74)	**0.002**
Underweight	25	(3.0%)	14	(8.1%)	39	(3.9%)	3.65	(1.83, 7.41)	**<0.001**
ASA – N(%)									
I	143	(21.3%)	30	(21.3%)	173	(21.3%)	1.00	–	–
II	523	(77.9%)	110	(78.0%)	633	(78.0%)	1.00	(0.64, 1.56)	0.991
III	5	(0.7%)	1	(0.7%)	6	(0.7%)	0.95	(0.11, 8.46)	0.966
Arterial Hypertension – N(%)									
No	219	(24.3%)	61	(31.3%)	280	(25.5%)			
Yes	683	(75.7%)	134	(68.7%)	817	(74.5%)	0.70	(0.50, 0.99)	**0.043**
Diabetes mellitus – N(%)									
No	669	(74.0%)	167	(85.6%)	836	(76.1%)			
Yes	235	(26.0%)	28	(14.4%)	263	(23.9%)	0.48	(0.31, 0.73)	**0.001**
Heart Disease – N(%)									
No	852	(94.2%)	185	(94.9%)	1037	(94.4%)			
Yes	52	(5.8%)	10	(5.1%)	62	(5.6%)	0.89	(0.44, 1.77)	0.732
Dyslipidemia – N(%)									
No	853	(94.4%)	187	(95.9%)	1040	(94.6%)			
Yes	51	(5.6%)	8	(4.1%)	59	(5.4%)	0.72	(0.33, 1.53)	0.389
Rheumatoid arthritis – N(%)									
No	858	(94.9%)	180	(92.8%)	1038	(94.5%)			
Yes	46	(5.1%)	14	(7.2%)	60	(5.5%)	1.45	(0.78, 2.70)	0.239
Anemia – N(%)									
No	632	(77.4%)	118	(68.2%)	750	(75.8%)			
Yes	185	(22.6%)	55	(31.8%)	240	(24.2%)	1.59	(1.11, 2.28)	**0.011**
Blood transfusion – N(%)									
No	777	(86.6%)	154	(79.0%)	931	(84.7%)			
Yes	127	(14.1%)	41	(21.0%)	168	(15.3%)	1,63	(1.10, 2.41)	0.015
Age (years) – median (IIQ)	67	(63–73)	70	(64–76)	68	(63–74)	–	–	0.001
Hematocrit (%)– median (IIQ)	39,0	(36.6–41.9)	38.2	(35.6–41.1)	38.9	(36.4–41.7)	–	–	0.012
Hemoglobin (g/dL) – median (IIQ)	12,9	(12.1–13.9)	12.9	(11.9–13.8)	12.9	(12.0–13.9)	–	–	0.113
White Blood Cell (cel./mm ^3^ ) – median (IIQ)	7300	(6300–8600)	5900	(4900–7700)	7200	(6000–8500)	–	–	<0.001
BMI (kg/m ^2^ ) – median (IIQ)	30,8	(27.5–34.7)	29.2	(26.0–32.7)	30.6	(27.3–34.5)	–	–	<0.001
Blood transfusion Volume (ml) – median (IIQ)	290	(256–513)	288	(261–511)	290	(257–512)	–	–	0.973

Age, underweight status, normal weight, and anemia were independent factors linked to a higher prevalence of malnutrition, while diabetes mellitus showed an inverse association ( Table 2 ). This analysis included 901 participants with complete data for all variables.

**Table 2:** Result of the multivariate logistic regression model of factors associated with malnutrition in patients ( *n* = 901) undergoing primary total knee arthroplasty

	Odds Ratio	95% CI	p
Age (years)	1.03	(1.01, 1.05)	0.014
BMI (OMS/OPAS)			
Obesity	1.00	–	–
Overweight	1.61	(0.99, 2.62)	0.057
Normal Weight	1.85	(1.21, 2.82)	0.005
Underweight	3.13	(1.50, 6.50)	0.002
Diabetes mellitus	0.60	(0.38, 0.96)	0.031
Anemia	1.55	(1.05, 2.28)	0.028
*Intercept*	*0.02*	*(0.00, 0.10)*	*0.000*

In assessing unfavorable outcomes after primary TKA, malnutrition ( *p* = 0.025) and transferrin levels ( *p* = 0.021) were associated with postoperative hospitalization time in bivariate analysis. However, other outcomes—early PJI, readmission for any cause, and readmission due to operative wound complications—did not correlate with nutritional status or serum markers ( Table 3 ). In the multivariate analysis, only anemia was independently associated with postoperative hospitalization duration ( Table 4 ). The bivariate analysis of factors related to hospitalization length is presented in Supplementary Table S3 .

**Table 3:** Analysis of malnutrition and nutritional serologic markers as a risk factor for unfavorable outcomes after primary total knee arthroplasty

	Early Periprosthetic Joint Infection				Hospital Readmission				Hospital Readmission for Wound Complications				Days from surgery to Hospital Discharge	
		OR	CI 95%	p		OR	CI 95%	p		OR	CI 95%	p	Median (IIQ)	p
Malnourished ( *n* = 195)	3 (1.5%)	0.93	0.17- 3.32	0.904	17 (8.7%)	0.91	0.49- 1.59	0.730	8 (4.1%)	0.70	0.28- 1.52	0.358	4 (3–5)	**0.025**
Nonmalnourisehd ( *n* = 904)	15 (1.7%)				86 (9.5%)				52 (5.8%)				3 (3–5)	
Albumin <3,5 g/dL ( *n* = 30)	1 (3.3%)	2.16	0.51–14.35	0.447	3 (10.0%)	1.15	0.22–3.81	0.825	2 (6.7%)	1.26	0.14–5.18	0.751	4 (3–6)	0.149
Albumin >3,5 g/dL ( *n* = 1403)	22 (1.6%)				124 (8.8%)				75 (5.4%)				3 (3–5)	
Transferrin <200mg/dL ( *n* = 84)	1 (1.2%)	0.66	0.02–4.28	0.689	5 (6.0%)	0.62	0.19–1.55	0.304	4 (4.8%)	0.88	0.23–2.48	0.816	4 (3–6)	**0.021**
Transferrin >200mg/dL ( *n* = 1065)	19 (1.8%)				99 (9.3%)				57 (5.4%)				3 (3–5)	
Lymphocytes <1.500 cell/mm ^3^ ( *n* = 205)	6 (2.9%)	1.51	0.51–3.68	0.358	22 (10.7%)	1.19	0.71–1.93	0.458	12 (5.9%)	1.1	0.54–2.07	0.752	4 (3–5)	0.055
Lymphocytes >1.500 cell/mm ^3^ ( *n* = 1783)	35 (2.0%)				163 (9.1%)				95 (5.3%)				3 (3–5)	

**Table 4:** Multivariate analysis of factors associated with primary total knee arthroplasty and hospital discharge interval

	Hazard Ratio	95% CI	p
Age (years)	0.995	(0.980, 1.000)	0.056
Anemia	0.843	(0.754, 0.941)	**0.002**
*Intercept*	*0.352*	*(0.248–0.499)*	*<0.001*

## Discussion

In this study, we found a laboratory-defined malnutrition prevalence of 17.7% among 1,099 patients undergoing primary TKA. Malnutrition was linked to advanced age, low and normal weight, and anemia, but inversely related to diabetes. No independent association was observed between malnutrition and PJI or noninfectious complications post-TKA.

The prevalence of hypoalbuminemia was lower than reported in the literature, with only 2.1% of patients meeting the malnutrition criterion. Nelson et al. (2015) 19 found that 4.2% of over 37,000 patients undergoing primary TKA in the USA had low serum albumin. In South Korea, Morey et al. (2016) 20 reported 7.1% malnutrition based on albumin levels. This variation may be due to geographic differences in dietary profiles, as global data show higher total and animal protein intake in Brazil compared with the USA and South Korea. 21

Using transferrin serum levels, malnutrition was identified in 7.3% of the sample. Huang et al. (2013) 22 found 6.6% malnutrition in patients undergoing elective arthroplasty. While their study linked malnutrition to higher complication rates, our findings differed. They also reported low overlap between albumin and transferrin, with 4.9% of malnourished patients showing both markers altered, similar to our study's 3.6%.

Total lymphocyte count was the most common malnutrition marker in our cohort (10.3%). This aligns with previous studies, such as Morey et al. (2016), 20 who found a 16% incidence. Overlap between lymphocyte and albumin markers was rare, occurring in just 1% of cases in our study and 7.7% in theirs. The lack of overlap among nutritional markers is notable, as malnutrition typically affects protein synthesis and immunity together, yet concurrent changes in albumin, transferrin, and lymphocytes were not observed here or in previous studies. 20 22

Our study found a small, yet statistically significant, association between malnutrition and advanced age, likely due to factors such as reduced appetite, food intake, comorbidities, polypharmacy, and functional decline. 23 Family isolation also plays a role, as increased dependence, loss of autonomy, and lack of home support negatively impact nutrition. 24 These issues were common in our population, often due to mobility loss from orthopedic conditions.

Anemia indicates poor nutritional status, especially in the elderly. 25 In our study, transferrin levels were used to assess malnutrition, but these vary based on anemia type: they increase in iron deficiency to optimize metal absorption and decrease in chronic disease along with that of other proteins. 26 Thus, the link between anemia and malnutrition may not be solely due to nutrient deficiencies but also chronic disease conditions associated with malnutrition. 27 Additionally, iron deficiency anemia can lower lymphocyte counts, explaining the independent association between anemia and malnutrition. 28

We found an association between malnutrition and lower BMI categories (“underweight” and “normal weight”), with higher risk compared with obese participants (BMI ≥ 30 kg/m ^2^ ). The prevalence of malnutrition in the underweight group was expected, as a BMI below 18.5 kg/m ^2^ is often a diagnostic criterion. However, even normal-weight individuals had higher rates of laboratory malnutrition. This may be due to osteoarthritis-related inactivity, leading to chronic conditions, lean mass loss, and fat replacement, contributing to malnutrition despite a normal BMI. In arthroplasty patients, normal-weight individuals showed a higher risk of malnutrition than overweight and obese patients (51% versus 27% and 32%, *p* = 0.0012 and 0.0023). 29 Age-related changes also support using the PAHO's BMI classification for the elderly, which better identifies underweight individuals at nutritional risk and is linked to higher mortality. 30

Interestingly, diabetes appears to be a protective factor against malnutrition. In lumbar spine surgery patients, insulin-dependent diabetes was linked to higher hypoalbuminemia rates, while non-insulin-dependent diabetes had similar rates to nondiabetics. 31 Studies on diabetes and malnutrition show mixed results. An Austrian survey of hospitalized and geriatric patients found diabetes to be a protective factor using the MUST score, nearing statistical significance (OR = 0.883, *p* = 0.06). 32 Conversely, diabetes was an independent risk factor for malnutrition in kidney carcinoma patients assessed by the SGA scale. 33 Most studies found no significant association between diabetes and malnutrition risk. 34 35 In our population, factors such as greater focus on dietary protein and stricter nutritional monitoring in high-risk diabetic patients may have contributed to better nutritional status. Additionally, diagnostic bias could play a role, as individuals with higher socioeconomic status may have better nutrition, greater healthcare access, and a higher likelihood of having or reporting a diabetes diagnosis.

Neither malnutrition nor serum marker levels were independent risk factors for complications after TKA. The only associations noted were between malnutrition and transferrin levels with postoperative length of stay, observed only in bivariate analysis, likely due to confounding factors like age and anemia. Fu et al. 13 found that hypoalbuminemia (albumin < 3.5 g/dL) was associated with total length of stay, but only in bivariate analysis. However, the independent link between anemia and malnutrition prevalence, along with increased postoperative length of stay, highlights that preoperative anemia is a modifiable yet often overlooked risk factor. 14

Several studies indicate that malnutrition increases the risk of both infectious and noninfectious complications after arthroplasty. In a study with 9,001 arthroplasty patients, those with albumin concentration < 3.5 g/dl were more likely to develop deep PJI (adjusted odds ratio 4.69, *p* < 0.001). 36 The infection rate in patients with low albumin (7.3%) was significantly higher than in our study (3.3%). This discrepancy may be due to the inclusion of hip arthroplasties, particularly urgent surgeries for proximal femur fractures, which carry a higher risk of early PJI compared with elective procedures. 37

Bohl et al. 14 identified serum albumin concentration as a potential risk marker for periprosthetic joint infection (PJI), perioperative complications, and readmission. They found an association between hypoalbuminemia, extreme BMI (<18.5 kg/m ^2^ and >40 kg/m ^2^ ), and advanced age (>70 years). These results were supported by Fu et al., 13 who reported higher septic and aseptic complication rates in patients undergoing TKA. However, Morey et al. 20 found no impact of malnutrition on infection rates or functional outcomes in 3,169 TKA patients, suggesting that nutritional assessment through laboratory parameters may not effectively stratify the risk of post-TKA infection.

A 2019 systematic review with meta-analysis evaluated 20 studies on the effects of laboratory-defined malnutrition in patients undergoing primary or revision total knee arthroplasty (TKA) and total hip arthroplasty (THA). Among the 11 studies involving over 100,000 patients with primary TKA, the combined prevalence of laboratory malnutrition was 11.6%. Only one study found no link between malnutrition and postoperative complications. The meta-analysis included eight comparable studies, which revealed a significant association between an albumin level < 3.5 g/dL and operative wound complications in arthroplasty patients (OR: 2.176, 95% CI: 1.916–2.471). However, the review did not address publication bias or article heterogeneity. Limitations included inconsistencies in malnutrition definitions, variations in assessed outcomes, and limited data on the timing of malnutrition marker collection. 38

This study has several limitations. We obtained analyzable results for albumin, transferrin, and lymphocytes in 68.9%, 55.2%, and 95.7% of the sample, respectively. Similar studies have reported 30% to 70% losses, a common limitation in this study design. 19 36 Despite the significant sample size, only 18 early PJI cases were identified, limiting the analysis of infection risk factors. The lack of other malnutrition assessments, such as nutritional questionnaires and body composition analysis, may have led to classification bias, misclassifying malnourished patients as eutrophic and vice versa. However, strengths include the focus on TKA, the use of the PAHO classification to assess nutritional risk by BMI in older adults, and the restriction of laboratory exam dates to six months before the procedure. Additionally, we utilized the three most recognized laboratory parameters for malnutrition—albumin, transferrin, and lymphocytes—enabling evaluation of each criterion's individual and combined effectiveness.

## Conclusions

The prevalence of altered lymphocyte count, serum albumin, and transferrin levels in patients undergoing TKA was 17.7%, with higher rates observed in older patients, anemic patients, and individuals with normal or low BMI. Identifying these groups could enhance preoperative management through nutritional interventions for partial or complete correction before surgery. Further studies with greater statistical power are needed to evaluate the association between malnutrition—regardless of the definition used—and adverse outcomes post-TKA.
