# Supplementary material for: Lymphocyte Count, Serum Albumin and Transferrin Levels in Patients Undergoing Total Knee Arthroplasty
Source: Rev Bras Ortop (Sao Paulo). 2025 Jul 25;60(2):s00451809529. doi: 10.1055/s-0045-1809529 (PMC12302328; doi:10.1055/s-0045-1809529)
Supplement: Supplementary file 1 — Supplementary Material [file 10-1055-s-0045-1809529_s2400329en.pdf]

Supplementary Table S1 Descriptive statistics of sample ($n = 2,080$)

	Frequency or Median	% or IQR
Gender		
Female	1,530	73.6%
Male	550	26.4%
BMI category WHO/PAHO ($n = 1,861$)		
Obesity	1,025	55.1%
Overweight	327	17.6%
Normal Weight	435	23.4%
Underweight	74	4.0%
ASA $n = 1,522$		
I	316	20.8%
II	1,194	78.4%
III	12	0.8%
Systemic arterial hypertension $n = 2,077$		
No	543	26.1%
Yes	1,534	73.9%
Diabetes mellitus		
No	1,596	76.7%
Yes	484	23.3%
Heart disease		
No	1,949	93.7%
Yes	131	6.3%
Dyslipidemia		
No	1,979	95.1%
Yes	101	4.9%
Rheumatoid Arthritis $n = 2,079$		
No	1,952	93.9%
Yes	127	6.1%
Anemia $n = 1,797$		
No	1,389	77.3%
Yes	408	22.7%
Early Periprosthetic Infection		
No	2,037	97.9%
Yes	43	2.1%
Readmission		
No	1,892	91.0%
Yes	188	9.0%
Readmission for local wound complication		
No	1,970	94.7%
Yes	110	5.3%
Age (years)	68	(63–74)
Hospital lenght stay (days)	5	(4–8)
Days from admission to surgery (days)	1	(1–3)
Days from surgery to discharge (days)	4	(3–5)

 © 2025. The Author(s).

Supplementary Table S1 (*Continued*)

	Frequency or Median	% or IQR
BMI (kg/m^2)	30.6	(27.1–34.5)
Blood transfusion volume (ml)	309	(269–563)

Abbreviations: ASA, American Society of Anesthesiologists risk score; BMI, Body mass index; IQR, interquartile range; PAHO, Pan American Health Organization; WHO, World Health Organization.

Supplementary Table S2 Descriptive statistics of laboratory markers of malnutrition ($n = 2,080$)

	Frequency or Median	% or IQR
Albumin <3.5 g/dL $n = 1,433$		
No	1,403	(97.9%)
Yes	30	(2.1%)
Lymphocytes <1.500 cells/mm^3 $n = 1,988$		
No	1,783	(89.7%)
Yes	205	(10.3%)
Transferrin <200 mg/dL $n = 1,149$		
No	1,065	(92.7%)
Yes	84	(7.3%)
Complete laboratory data		
No	981	(47.2%)
Yes	1,099	(52.8%)
One or more malnutrition criteria $n = 1,099$		
No	904	(82.3%)
Yes	195	(17.7%)
Two or more malnutrition criteria $n = 1,099$		
No	1,082	(98.5%)
Yes	17	(1.5%)
Albumin (g/dL) $n = 1,433$	4.0	(3.8–4.2)
Lymphocytes (cells/mm^3) $n = 1,988$	2,280.0	(1863.0–2820.0)
Transferrin (mg/dL) $n = 1,149$	249.0	(225.0–278.0)

Supplementary Table S3 Bivariate analysis for hospital stay length

	Duration of Hospital Stay Following Primary TKA Surgery (Days)	p-value
	Mediana (IIQ)	
Albumin <3.5 g/dL		
Yes	4 (3–6)	0.149
No	3 (3–5)	
Transferrin <200mg/dL		
Yes	4 (3–6)	**0.021**
No	3 (3–5)	
Lymphocytes <1.500 cells/mm3		
Yes	4 (3–5)	0.055
No	3 (3–5)	

(*Continued*)

 © 2025. The Author(s).

Supplementary Table S3 (*Continued*)

	Duration of Hospital Stay Following Primary TKA Surgery (Days)	p-value
	**Mediana (IIQ)**	
Gender		
Female	4 (3–5)	0.336
Male	3.5 (3–5)	
ASA		
I ($n=316$)	3 (2.5–4)	**0.002**
II ($n=1194$)	4 (3–5)	
III ($n=12$)	5 (3.5–7)	
Age		
<65 ($n=689$)	3 (3–4)	<**0.001**
≥ 65 e <72 ($n=673$)	4 (3–5)	
≤ 72 ($n=718$)	4 (3–5)	
Blood Tranfusion		
Yes	6 (4–9)	<**0.001**
No	3 (3–4)	
Early Periprostetic Infecction		
Yes	4 (3–6)	0.078
No	4 (3–5)	
Systemic arterial hypertension		
Yes	4 (3–5)	0.253
No	3 (3–5)	
Diabetes mellitus		
Yes	4 (3–5)	0.663
No	3 (3–5)	
Heart disease		
Yes	4 (3–5)	**0.014**
No	4 (3–5)	
Precordial pain		
Yes	3 (2–7)	0.617
No	4 (3–5)	
Dyslipidemia		
Yes	3 (3–5)	0.058
No	4 (3–5)	
Stroke		
Yes	5 (3–5)	0.127
No	4 (3–5)	
Tumor		
Yes	3 (3–6)	0.591
No	4 (3–5)	
Malnutrition		
Yes	4 (3–5)	**0.025**
No	3 (3–5)	

 © 2025. The Author(s).

Supplementary Table S3 (*Continued*)

	Duration of Hospital Stay Following Primary TKA Surgery (Days)	p-value
	**Mediana (IIQ)**	
IMC		
Underweight	4 (3–5)	0.155
Normal Weight	4 (3–5)	
Overweight	3 (3–4)	
Obesity	3 (3–5)	
Anemia		
Yes	4 (3–6)	<**0.001**
No	3 (3–5)	

 © 2025. The Author(s).
